# Factors associated with the modulation of pain by visual distortion of body size

**DOI:** 10.3389/fnhum.2014.00137

**Published:** 2014-03-20

**Authors:** Michihiro Osumi, Ryota Imai, Kozo Ueta, Hideki Nakano, Satoshi Nobusako, Shu Morioka

**Affiliations:** ^1^Department of Neurorehabilitation, Graduate School of Health Science, Kio UniversityNara, Japan; ^2^Neurocognitive Rehabilitation Center, Setsunan General HospitalOsaka, Japan; ^3^Japan Society for the Promotion of ScienceTokyo, Japan

**Keywords:** pain, body representation, distortion of body size, mirror visual feedback, illusion, body consciousness

## Abstract

Modulation of pain using visual distortion of body size (VDBS) has been the subject of various reports. However, the mechanism underlying the effect of VDBS on pain has been less often studied. In the present study, factors associated with modulation of pain threshold by VDBS were investigated. Visual feedback in the form of a magnified image of the hand was provided to 44 healthy adults to examine changes in pain. In participants with a higher pain threshold when visual feedback of a magnified image of the hand was provided, the two-point discrimination threshold decreased. In contrast, participants with a lower pain threshold with visual feedback of a magnified image of the hand experienced unpleasant emotions toward the magnified image of the hand. Interestingly, this emotional reaction was strongly associated with negative body consciousness in several subjects. These data suggested an analgesic effect of visual feedback in the form of a magnified image of the hand is only when tactile perception is vivid and the emotional reaction toward the magnified image is moderate. The results also suggested that negative body consciousness is important for the modulation of pain using VDBS.

## INTRODUCTION

Pain is a conscious experience. Pain is influenced not only by peripheral organs but also by anxiety, fear, attention, and expectancy (Tracey and Mantyh, 2007). Therefore, various kinds of stimuli (emotional, visual, auditory) modulate the perception of pain. For example, unpleasant sounds increase pain intensity (Drummond and Willox, 2013), and odor valence increases the unpleasant characteristics of the pain (Villemure et al., 2003). Furthermore, simultaneous administration of visual and pain stimuli alters the perception of pain. Meagher et al. (2001) reported that viewing expressions of fear and disgust decreases the pain threshold. Conversely, experimental pain is decreased when a subject views images of a romantic partner (Younger et al., 2010; Eisenberger et al., 2011).

In a recent study, the analgesic effects of body visualization notably received attention. Longo et al. (2009, 2012) reported decreases in pain intensity, unpleasant emotions associated with pain as a result of visual information about the body provided concurrently with pain stimulation. In a clinical research study of patients with complex regional pain syndrome (CRPS), tactile discrimination training involved visualization of the body reflected in a mirror. A more analgesic effect was observed using this technique than using tactile discrimination training alone (Moseley and Wiech, 2009). More recently, the analgesic effect for chronic lower back pain was better when repeated visual feedback of the body during movement of the lumbar spine was used than when no visual feedback was used (Wand et al., 2012). Therefore, rehabilitation through motor tasks or perception training combined with body visualization is preferable for the treatment of pain.

Furthermore, some reports suggest that the perception of pain is modulated when subjects view distorted images of their body size. Moseley et al. (2008a) reported an increase in pain intensity and swelling during visualization of a magnified image of the affected body part during movement in patients with CRPS. This study was the first, as per our knowledge, to report pain modulation through visual distortion of body size (VDBS). However, the results of other studies contradict this finding (Ramachandran et al., 2009; Mancini et al., 2011; Diers et al., 2013). In the case of patients with phantom limb pain, no change in pain intensity was observed when a magnified mirror image of the intact limb was provided; however, visualization of a reduced mirror image decreased pain intensity (Ramachandran et al., 2009). Mancini et al. (2011) reported that the analgesic effect was better with visual feedback using magnification than with visual feedback without magnification in healthy subjects. Furthermore, in patients with chronic low back pain, although viewing actual-sized images of their own trunks on a monitor decreased pain intensity, no difference was detected when magnified and reduced images were presented (Diers et al., 2013). Therefore, reports about VDBS for pain modulation vary in their results.

Factors associated with the modulation of pain by VDBS must be identified. In a clinical setting for the treatment of patients with chronic pain, the effect of this technique may vary considerably from one individual to another; pain may even increase for some patients. In this study, factors associated with variations in the effects of visualizing magnified images of body parts were examined.

With regard to the effects of viewing magnified body images, some reports have documented strengthening of somatosensory perception (Kennett et al., 2001; Taylor-Clarke et al., 2004; Longo and Haggard, 2011). In contrast, some reports have documented that viewing of magnified body images can trigger unpleasant emotions in individuals suffering from negative body consciousness and activate areas of the brain that are also active during pain, for example, the prefrontal cortex, anterior cingulate gyrus, or the insular cortex (Friederich et al., 2010; Miyake et al., 2010; Mohr et al., 2011; Spangler and Allen, 2012). Therefore, to research factors associated with pain modulation by VDBS, we designed experiments from two different perspectives: change in “somatosensory perception” and change in “emotion.”

According to the literature, we hypothesized that individuals with a decreased pain threshold will feel unpleasant on viewing their magnified body image, that individuals with an increased pain threshold will experience a more vivid somatosensory perception on viewing their magnified body image, and that changes in emotion and somatosensory perception induced by viewing a magnified body image is related to body consciousness. Using a magnified mirror visual feedback technique, we collected quantitative data on changes in the two-point discrimination threshold (TPD) and in self-rated feelings toward the affected body part. In addition, qualitative data about emotional responses were obtained using open questions such as “How did that feel?” and “Were you aware of any changes in either limb?” Finally, we studied the relationship between changes in emotion and somatosensory perception after exposure to magnified mirror visual feedback and with regard to the body consciousness of each participant.

## MATERIALS AND METHODS

### PARTICIPANTS

A total of 44 healthy right-handed students (17 males, 27 females; mean age, 21.6 years; SD, 1.7) participated in this study. They were recruited for the experiment from the campus of Kio University. The study protocol conformed to the Declaration of Helsinki. All participants were informed at the start of the study that they could discontinue participation at any time during the experiments. We explained the details of the experimental procedure but not the purpose of the experiment in order to avoid bias in results. Before participating, subjects provided written informed consent. This study was approved by the ethics committee of Kio University Health Science Graduate School (approval number: H24-19).

### THERMAL STIMULUS DEVICE AND MEASUREMENT OF PAIN THRESHOLD

Thermal stimulation of the dorsum of the left hand just proximal to the knuckle of the index finger (first metacarpal space) was delivered by a pain thermometer (UDH-105, Unique Medical, Japan). The probe measured 20 mm in diameter and contacted the skin at the measurement site. Pain threshold was estimated using the method of limits (Yarnitsky et al., 1995). The probe temperature was increased from normal skin temperature (constant 32°C maintained for 20 s) at 1°C per second. The pain threshold was identified when participants first perceived the stimulation to be painful. For safety, the maximum temperature was limited to 50°C. In addition, to avoid habituation to contact heat pain stimulation, pain stimulation was administered to different sites during an experimental trial (the center of the dorsum of the left hand) before the experimental procedure.

### PROCEDURE

**FIGURE 1:**
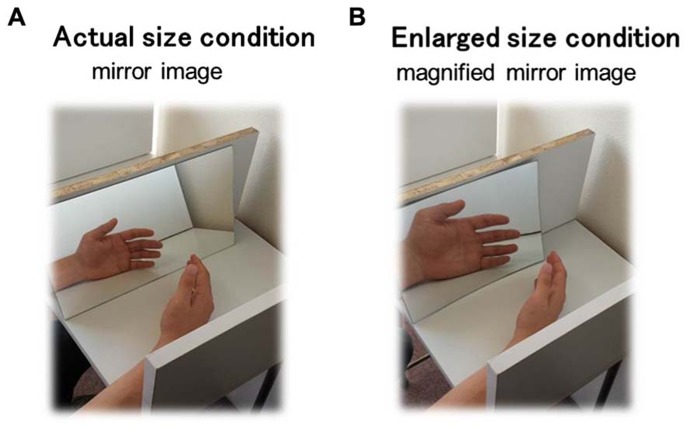
**Schema of the experimental paradigm setup in this study.** Subjects whose pain threshold and two-point discrimination threshold were measured during visualization of a mirror image of the hand under an actual size condition **(A)** and an enlarged size condition **(B)**.

The magnifying mirror-box technique (Mancini et al., 2011) was used in this study to allow visualization of the magnified affected body part, in this case, the left hand. Participants were instructed to sit at a table with the left arm on the table, where it was reflected in a mirror aligned with the sagittal plane. The left arm was positioned in such a way that it did not allow visualization of the actual left hand. In this position, the participants do not view the experimental hand, but rather a reflection of the unstimulated hand. Therefore, they think that the hand reflected in the mirror is their actual left hand (**Figure 1**). The experiment was conducted under two conditions. For visualization of the magnified mirror image of the hand (enlarged size condition), a concave mirror with 2× magnification was used. For visualization of the unmagnified mirror image of the hand (actual size or control condition), a normal mirror was used.

First, to allow time to adapt to visualization of the mirror image of the hand, participants were instructed to look in the mirror while moving their hands bilaterally at a self-selected rate for 10 min in each condition. Then, the TPD was measured with visualization of the mirror image of the dorsum of the left hand. TPD was performed reference from Moberg (1990). A mechanical caliper with a precision of 1 mm was applied until the very first blanching of the skin appeared around the prongs. Testing commenced with 30 mm between the two points of the caliper, and the distance was decreased in 1-mm increments until the subject was able to perceive one point instead of two. They were instructed to provide a verbal indication when two points were felt. The smallest distance was designated as the point of TPD.

Third, the pain threshold of the left hand behind the mirror was measured. A fake thermode probe was simultaneously applied to the right hand at the location corresponding to that on the left hand. In this experiment, the pain threshold was measured four times at 1-min intervals, and the mean temperature was designated as the pain threshold.

In addition to this procedure, three questionnaires associated with the illusion of ownership of the mirror image of the hand were distributed to participants. Three items on this questionnaire were adopted from the research of Longo et al. (2009) and Eisenberger et al. (2011): (1) It felt like I was looking directly at my hand rather than at a mirror image, (2) It felt like the hand I was looking at was my hand, (3) Did it seem like the hand you saw was a right hand or a left hand? One questionnaire item was associated with the feeling toward the mirror image of the hand (feelings toward the hand), and (4) What is your impression of the hand you see? Participants rated their agreement with items 1 and 2 on a 7-point Likert scale ranging from +3 (strongly agree) to –3 (strongly disagree). For item 3, responses were scored on a scale from –100 (strong feeling of viewing the right hand) to 100 (strong feeling of viewing the left hand). For item 4, responses were scored on a scale from –100 (strongly undesirable) to 100 (strongly desirable). The conditions were randomly ordered to avoid bias.

In the enlarged size condition only, to examine qualitative changes in subjective perception of and emotional reaction to the magnified mirror image of the hand, participants were asked a series of open questions adopted from another study: “How did that feel?” followed by the further prompt, “Were you aware of any changes in either limb?” (McCabe et al., 2005, 2007). Participants were asked these questions after we acquired measurements of TPD and pain threshold. No specific direct inquiry was made about possible sensory changes to prevent leading of the subjects and avoid inducing a possible source of bias.

### SELF-REPORTED QUESTIONNAIRES

To investigate the body consciousness of each participant, the body shape questionnaire (BSQ) and body attitudes questionnaire (BAQ) were used (Cooper et al., 1987; Ben-Tovim and Walker, 1991). The BSQ is used to estimate the obsessiveness of the shape and appearance of one’s body. High scores on this questionnaire indicate strong obsessiveness to the shape and appearance of one’s body. The BAQ is used to estimate a subject’s thought about the body. High scores on this questionnaire indicate negative thoughts about the body. BSQ and BAQ have been used in various countries to study healthy individuals as well as those suffering from eating disorders, with satisfactory reliability and validity (Traverso et al., 2000; Wade et al., 2003; Ghaderi and Scott, 2004; Burgess et al., 2006; Smeets et al., 2009; Akdemir et al., 2012; Welch et al., 2012).

### STATISTICAL ANALYSIS

First, to ensure that participants were convinced that the mirror image represented their own hand, their agreement or disagreement with the three questionnaire items was evaluated using *t*-tests that compared the score for each item and condition with 0. To determine whether the illusion of body ownership differed between conditions, agreement or disagreement with the three questionnaire items was evaluated using *t*-tests that compared the scores for actual and enlarged sizes for each item.

To investigate factors associated with the change in pain threshold under the Enlarged size condition, participants were grouped into high or low threshold groups according to the variations in pain thresholds among conditions. Subjects in the high threshold group exhibited a higher pain threshold under the enlarged size condition than under the actual size condition (*n* = 23). Subjects in the low threshold group exhibited a lower pain threshold under the enlarged size condition than under the actual size condition (*n* = 21).

Qualitative data generated from subjects’ responses to the open questions about the magnified mirror image of the hand were tabulated in Microsoft Excel and analyzed using content analysis (McCabe et al., 2005, 2007). Subjects were each allocated a unique code, and responses to the open questions were typed against the individual’s code under the relevant stage in the protocol. Then, the number of individuals allocated each code by content analysis was compared between the high and low threshold groups using Fisher’s exact test.

TPD and feelings toward the hand were analyzed using two-way repeated-measures ANOVA for two binary factors, group (high and low threshold groups) and condition (actual and enlarged size conditions). The Bonferroni method was used for *post hoc* comparisons.

To investigate the relationship between the variations in feelings toward the hand, TPD, and body consciousness, the Spearman correlation coefficient was used to analyze correlations among BSQ and BAQ scores and variations in feelings toward the hand and TPD. Variations in feelings toward the hand and TPD were calculated by subtracting the value for the enlarged size condition from that for the actual size condition.

All results are reported as means ± standard deviations. Statistical analysis was performed with SPSS ver. 17.0 (SPSS, Chicago, IL, USA). An alpha level of 5% was considered as statistically significant.

## RESULTS

### PAIN THRESHOLD

In the high threshold group, the pain threshold under the actual size and enlarged size conditions was 44.66 ± 2.58°C and 46.03 ± 2.95°C, respectively. In the low threshold group, the pain threshold under these conditions was 44.27 ± 2.09°C and 43.01 ± 2.26°C, respectively. To validate the group assignments, we compared the pain thresholds of the two groups using Student’s unpaired *t*-test for each condition. There were no significant differences in pain threshold between groups under the actual size condition (*t* = –0.67, *p* = 0.50). In contrast, there were significant differences (*t* = –3.77, *p* < 0.001) in pain threshold between groups under the Enlarged size condition.

### DEGREE OF ILLUSION UNDER EACH CONDITION

Under the actual size condition, scores for the illusion of body ownership were as follows: item 1, 1.59 ± 1.22; item 2, 2.11 ± 0.86; and item 3, 57.72 ± 34.81. Under the actual size condition, for all three items, the actual size mirror produced the illusion of body ownership with the following scores: item 1, *t* = 8.61, *p* < 0.001; item 2, *t* = 16.14, *p* < 0.001; and item 3, *t* = 11.01, *p* < 0.001. Under the enlarged size condition, scores for the illusion of body ownership were as follows: item 1, 0.91 ± 1.44; item 2, 1.20 ± 1.45; and item 3, 43.29 ± 33.74. Under the enlarged size condition, for all three items, the enlarged mirror produced the illusion of body ownership with the following scores: item 1, *t* = 4.18, *p* < 0.001; item 2, *t* = 5.49, *p* < 0.001; and item 3, *t* = 8.51, *p* < 0.001. However, the score for the illusion of body ownership under the actual size condition was higher than that under the enlarged size condition for all items: item 1, *t* = 3.32, *p* = 0.002; item 2, *t* = 3.78, *p* < 0.001; and item 3, *t* = 2.15, *p* = 0.03.

### QUALITATIVE DATA UNDER THE ENLARGED SIZE CONDITION

**Table 1** shows the code compiled from responses to open questions about the magnified mirror image of the hand and the number of subjects allocated to each code. More participants in the high threshold group responded that they felt nothing special. In the low threshold group, more participants responded that they felt unpleasant emotions (*p* < 0.05).

**Table 1:** Response frequencies for magnified mirror image of the hand in both the high threshold and low threshold groups.

	High threshold group (*n* = 23)	Low threshold group (*n* = 21)	*p*-value
Lightweight	3 (6.8%)	1 (2.3%)	0.33
Strength	4 (9.1%)	1 (2.3%)	0.20
Vaguely unpleasant	1 (2.3%)	1 (2.3%)	0.73
Unpleasant with swelling	2 (4.5%)	6 (13.6%)	0.09
Unpleasant with asymmetry	0 (0%)	2 (4.5%)	0.22
Unpleasant with large	1 (2.3%)	8 (18.1%)	0.01
Nothing special	12 (27.2%)	2 (4.5%)	0.01

*Significant at *p* < 0.05. The percentage of subjects allocated to each code (*n* = 44) is indicated in parentheses.*

### FEELINGS TOWARD THE MIRROR IMAGE OF THE HAND

**FIGURE 2:**
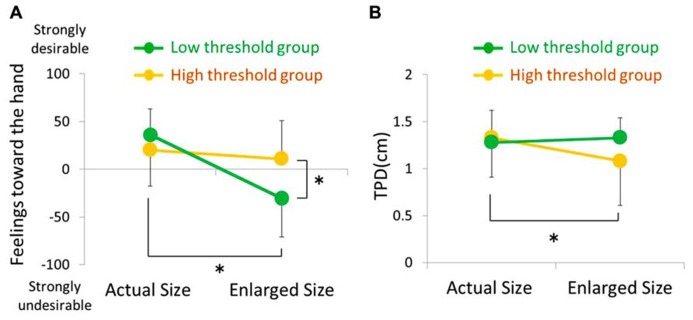
**Feelings toward the hand **(A)** and TPD **(B)** under actual size and enlarged size conditions in both the high threshold (orange bar) and low threshold (green bar) groups.** Error bar is SD.

Statistical analysis using two-way repeated ANOVA showed a significant main effect of condition (*F* = 23.51, *p* < 0.001), but not of group (*F* = 2.54, *p* = 0.115). There was a significant interaction between condition and group (*F* = 13.35, *p* < 0.001; **Table 2**; **Figure 2A**). *Post hoc* tests indicated a significant difference between the actual size and enlarged size conditions in the low-threshold group (*p* < 0.001) but not in the high-threshold group (*p* = 1.336). In addition, a significant difference was also observed between the high-threshold and low-threshold groups under enlarged size conditions (*p* = 0.006) but not under actual size conditions (*p* = 0.437). Therefore, compared with subjects in the high threshold group, subjects in the low threshold group had a more negative impression of the magnified mirror image of the hand under the enlarged size condition than under the actual size condition.

**Table 2:** Comparison ofTPD and Feeling toward the hand under actual size and enlarged size conditions between the high threshold and low threshold groups.

	Actual size condition	Enlarged size condition	Group × Condition	
			*F*-value	*p*-value
Feelings toward the hand	High threshold group	20.09 (26.82)	10.74 (40.41)	13.35	<0.001
	Low threshold group	36.19 (37.96)	–30.28 (40.06)		
TPD	High threshold group	1.33 (0.29)	1.08 (0.21)	4.05	0.04
	Low threshold group	1.28 (0.37)	1.33 (0.47)		

*Values are means (SD). Significant at*p* < 0.05.*

### TWO-POINT DISCRIMINATION THRESHOLD

Statistical analysis using two-way repeated ANOVA did not show a significant main effect of condition (*F* = 1.72, *p* = 0.18) or group (*F* = 1.82, *p* = 0.19). However, a significant interaction between condition and group (*F* = 4.05, *p* = 0.04) was detected (**Table 2**; **Figure 2B**). Furthermore, *post hoc* tests indicated a significant difference between actual and enlarged size conditions in the high-threshold group (*p* < 0.001), but not in the low-threshold group (*p* = 1.453). Moreover, *post hoc* tests did not report any significant difference between the high-threshold and low-threshold groups under enlarged size conditions (*p* = 0.111) or actual size conditions (*p* = 2.503). Therefore, subjects in the high threshold group experienced more vivid somatosensory perception under the enlarged size condition than actual size condition.

### CORRELATION ANALYSIS

**FIGURE 3:**
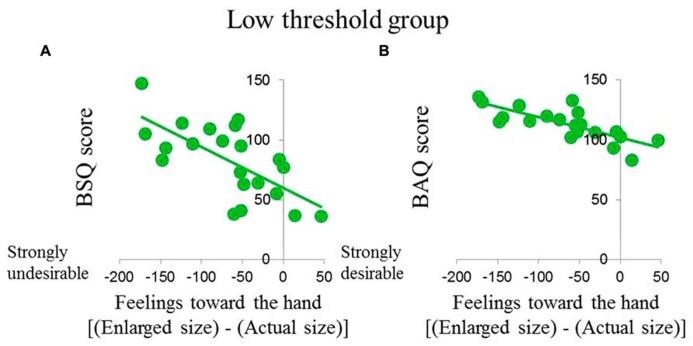
**Correlations among **(A)** BSQ score, **(B)** BAQ score, and change in feelings toward the hand in the low threshold group.** Data indicate significant correlations among parameters (*p* < 0.01).

**Table 3** shows the results of correlation analysis of variations in feelings toward the hand, TPD (actual size condition value subtracted from the enlarged size condition value), and BSQ and BAQ scores. A significant negative correlation was found between variations in feelings toward the hand and BAQ scores in both groups (*p* < 0.05). In contrast, a significant negative correlation between variations in feelings toward the hand and BSQ scores was found only in the low threshold group (*p* < 0.001; **Figure 3**).

**Table 3:** Correlation among BSQ score, BAQ score, variation of Feelings toward the hand, and TPD in both the high threshold and low threshold groups.

	Feelings toward the hand, *r* (*p*) (enlarged size–actual size)	TPD, *r* (*p*) (enlarged size–actual size)
High threshold group	BSQ	–0.28 (0.19)	0.19 (0.36)
	BAQ	–0.44 (0.03)^*^	0.26 (0.21)
Low threshold group	BSQ	–0.67 (<0.001)^**^	0.33 (0.13)
	BAQ	–0.77 (<0.001)^**^	0.31 (0.17)

* *p* < 0.05, **p < 0.01, *r*: Spearman's rank correlation coefficient. BSQ, body shape questionnaire; BAQ, body attitudes questionnaire.

## DISCUSSION

In the present study, research factors associated with the modulation of pain by visualization of a magnified image of the hand, changes in emotional reaction and somatosensory perception were compared between groups with high and low pain thresholds. The number of subjects who reported “unpleasant with large” on visualizing a magnified image of the hand was greater in the latter group than in the former group (**Table 1**). In addition, these subjects had a more negative impression of the magnified image of the hand than of the hand visualized at its actual size (**Table 2**; **Figure 2A**). On the other hand, the number of subjects who provided responses like “nothing special” during visualization of a magnified image of the hand was greater in the high threshold group than in the low threshold group (**Table 1**). This was also true for vividness of somatosensory perception (**Table 2**; **Figure 2B**). Therefore, in the low threshold group only, subjects with strong obsessiveness toward the shape and appearance of their own bodies had a negative impression of the magnified mirror visual feedback (**Figure 3**).

### FACTORS ASSOCIATED WITH INCREASED PAIN ON VISUALIZATION OF A MAGNIFIED MIRROR IMAGE OF THE HAND

In the present study, the increased pain on visualization of a magnified mirror image of the hand was associated with a negative emotional reaction (**Table 1**; **Figure 2A**). Pain is modified by expectations, context, and other cognitive and affective processes (Koyama et al., 2005; Brown et al., 2008; Atlas et al., 2010). For example, humans feel pain more strongly than they feel the actual pain stimulation when they are told that the pain will be strong (Koyama et al., 2005). The analgesic effect of visualizing the body is reportedly obstructed in contexts that elicit negative emotional reactions (Höfle et al., 2010, 2012; Martini et al., 2013). Höfle et al. (2012) reported an increase in pain intensity in contexts where fear of pain was elicited despite viewing the body. Martini et al. (2013) used a virtual reality system and found that viewing the arm turning red in association with injury decreased the pain threshold. In addition, Moseley et al. (2012) reported that the implicit perception of threat to body tissues caused increased pain and swelling by visualization of a magnified mirror image of the hand in patients with CRPS. Added to which, changes in pain due to bias and context may be similar to changes in pain due to negative emotions about the body. For example, for people with feelings of dislike about their bodies, the autonomic nervous response is overactivated when a pinprick is applied to a disliked body part (Brang et al., 2008). From these reports, previous research has suggested that pain may be increased by negative emotions about the body. In the present study, participants in the low threshold group had a spontaneous negative reaction to the magnified image of the hand, which elicited negative emotions about the body. Therefore, the pain threshold may have decreased accordingly.

### FACTORS ASSOCIATED WITH DECREASED PAIN ON VISUALIZATION OF A MAGNIFIED MIRROR IMAGE OF THE HAND

In the present study, decreased pain on visualization of a magnified mirror image of the hand was associated with more vivid perception (**Figure 2B**). Several studies have reported similar results; for example, visualizing a magnified mirror image of the hand decreased TPD in many subjects (Kennett et al., 2001; Taylor-Clarke et al., 2004; Longo and Haggard, 2011). These studies indicate that the experience of viewing one’s magnified body image contributes to the vividness of somatosensory perception. In contrast, TPD increases in patients with CRPS, chronic low back pain, and osteoarthritis (Vartiainen et al., 2009; Luomajoki and Moseley, 2011; Stanton et al., 2013). Therefore, somatosensory perceptual vividness in patients with chronic pain is decreased. Indeed, amelioration of pain in patients with chronic pain is associated with increased somatosensory perceptual vividness by tactile discrimination training (Barker et al., 2008; Moseley et al., 2008b; Moseley and Wiech, 2009). Therefore, increased somatosensory perceptual vividness may be associated with an analgesic effect. In the present study, the pain threshold of participants may have been higher because participants in the high threshold group experienced more somatosensory perceptual vividness during visualization of the magnified image of the hand.

### RELATIONSHIP WITH BODY CONSCIOUSNESS

In the present study, subjects with higher BAQ scores in both groups had stronger negative impressions toward the magnified mirror image of the hand (**Table 3**). The BAQ score is indicative of thoughts about the body, such as degree of health, enchantment (Ben-Tovim and Walker, 1991). Therefore, people with more negative body consciousness reported that felt stronger unpleasant emotions about the magnified mirror image of the hand. In a study of subjects with eating disorders and negative body consciousness, magnified images of the entire body elicited unpleasant reactions (Friederich et al., 2010; Miyake et al., 2010; Mohr et al., 2011; Spangler and Allen, 2012). In the present study, although only the hand was subjected to painful stimulation, a similar relationship was observed between negative body consciousness and unpleasant emotional reactions during visualization of a magnified image of the affected body part. Interestingly, a higher BSQ score was associated with a stronger negative impression toward the magnified mirror image of the hand only in the low threshold group. BSQ scores are indicative of the obsessiveness of the shape and appearance of one’s body (Cooper et al., 1987). Eshkevari et al. (2012) reported that individuals who obsess strongly about their appearance possess heightened sensitivity to visual information and increased perceptual plasticity about their bodies. Furthermore, Ainley and Tsakiris (2013) reported that self-objectification accounts for the poor interoceptive awareness. However, there are no available data, to our knowledge, that define the relationships among personality, emotional reaction to a magnified image of the body, and changes in pain perception induced by visualization of a magnified image of the body. Furthermore, distortion of subjective body size is one of the factors that influences chronic pain (Moseley, 2005; Lewis et al., 2007; Peltz et al., 2011; Bailey et al., 2013). For example, Moseley (2005) were the first to report that patients with CRPS estimated their own affected hand to be larger than it actually was. However, no research has been conducted on the psychological features associated with pain on viewing a magnified image of the body. In contrast, the present study reveals that the strong obsessiveness of the shape and appearance of one’s body was related to the strong unpleasant emotional reaction to a magnified image of the body only in individuals with increased pain induced by viewing a magnified image of their body. The results of the present study offer some insight into these features. Pain exacerbation during VDBS may be related to the obsessiveness of the shape and appearance of one’s body. Therefore, careful attention is required during application of this technique to the rehabilitation of patients with strong obsessiveness.

Many studies have reported on the effects of visualization of the magnified image of the body on pain. The results of these reports have varied. Some reported decreased pain (Mancini et al., 2011), others reported increased pain (Moseley et al., 2008a), and yet others reported no change (Ramachandran et al., 2009; Diers et al., 2013). The present study showed that various factors may be associated with these effects. Subjects may have gained relief from pain because of their vivid somatosensory perceptions during visualization of the magnified mirror image, while others may have experienced greater pain and negative emotions toward the magnified image of the hand. In addition, the importance of considering body consciousness was emphasized in this study. These results may be useful when applying the technique of VDBS in a clinical setting for patients with chronic pain. Recent reports have shown the positive effects of perception or motor training combined with the visual feedback of the body to chronic pain (Moseley and Wiech, 2009; Wand et al., 2011, 2012; Moseley and Flor, 2012). With regard to perception or motor training combined with visual feedback, the effects of vividness of somatosensory perception or decrease in pain by viewing the body was utilized (Serino et al., 2007; Longo et al., 2009). This study showed that pain decreased in individuals with vivid somatosensory perception on viewing a magnified image of their body. Therefore, the magnified mirror visual feedback technique combined with motor and perceptual training may provide an analgesic effect only if negative emotions are not elicited. However, more research is required on patients with chronic pain.

This study had several limitations. First, changes in emotion were measured during visualization of the magnified mirror image of the hand using a subjective battery of questions. Changes in automatic nerve and pain matrix activity were not directly measured. Therefore, future research including measurement of skin conductance and functional magnetic resonance imaging or electroencephalography must be conducted. Second, although TPD was measured as the change in perception during visualization, somatotopic representation in the primary somatosensory cortex was not measured. Therefore, research into plastic change and somatotopic representation in the primary somatosensory cortex must be performed using functional magnetic resonance imaging. Third, the effects of visualization of the magnified image of the body in patients with chronic pain must be investigated. Fourth, we did not show subjects an enlarged image of the hand using photographs, for example. We did show them a magnified mirror image of an enlarged hand because there were difficult subjects who felt like looking at their left hand. Fifth, we only used the left hand as the experimental hand. Sixth, we did not compare TPD when the body was viewed using TPD with the eyes closed. Seventh, although bias was minimalized as much as possible, there were sources of bias. Because the TPD values in this study were smaller than those published previously, we did not conduct a blind assessment or include a manipulation check. Eighth, we only evaluated pain threshold as an objective index. Although the evaluation of pain intensity using a rating scale can be informative, it was difficult to evaluate both because of mutual interference. Ninth, because subjects in this experiment were college students, the results cannot be generalized to the rest of the population. Ten, although participants felt the mirror image was their actual left hand in both of actual and enlarged size condition, the degree of illusion of body ownership under the actual size condition was stronger than that under the enlarged size condition.

This present study is the first, to our knowledge, to investigate the factors associated variability in the effects of VDBS on pain modulation. We found that VDBS increased pain to an unpleasant level that was related to the subject’s obsession with their body shape and appearance. In contrast, vividness of somatosensory perception decreases the perception of pain. In conclusion, although VDBS is useful for the management of pain, its limitations must be taken into account.

### Conflict of Interest Statement

The authors declare that the research was conducted in the absence of any commercial or financial relationships that could be construed as a potential conflict of interest.

## References

[B1] Ainley V. Tsakiris M. (2013). Body conscious? Interoceptive awareness, measured by heartbeat perception, is negatively correlated with self-objectification. *PLoS ONE* 8:e55568 10.1371/journal.pone.0055568 PMC3565964 23405173

[B2] Akdemir A. Inandi T. Akbas D. Karaoglan Kahilogullari A. Eren M. Canpolat B. I. (2012). Validity and reliability of a Turkish version of the body shape questionnaire among female high school students: preliminary examination. *Eur. Eat Disord. Rev.* 20 114–115 10.1002/erv.1106 21953701

[B3] Atlas L. Y. Bolger N. Lindquist M. A. Wager T. D. (2010). Brain mediators of predictive cue effects on perceived pain. *J. Neurosci.* 30 12964–12977 10.1523/JNEUROSCI.0057-10.2010 20881115 PMC2966558

[B4] Bailey J. Nelson S. Lewis J. McCabe C. S. (2013). Imaging and clinical evidence of sensorimotor problems in CRPS: utilizing novel treatment approaches. *J. Neuroimmune Pharmacol.* 8 564–575 10.1007/s11481-012-9405-9 23054370

[B5] Barker K. L. Elliott C. J. Sackley C. M. Fairbank J. C. (2008). Treatment of chronic back pain by sensory discrimination training. A phase I RCT of a novel device (FairMed) vs. TENS. *BMC Musculoskelet. Disord.* 9:97 10.1186/1471-2474-9-97 PMC2443795 18588702

[B6] Ben-Tovim D. I. Walker M. K. (1991). The development of Ben–Tovim walker body attitudes questionnaire (BAQ), a new measure of women's attitudes towards their own bodies. *Psychol. Med.* 21 775–784 10.1017/S0033291700022406 1946865

[B7] Brang D. McGeoch P. D. Ramachandran V. S. (2008). Apotemnophilia: a neurological disorder. *Neuroreport* 19 1305–1306 10.1097/WNR.0b013e32830abc4d 18695512

[B8] Brown C. A. Seymour B. Boyle Y. El-Deredy W Jones A. K. P. (2008). Modulation of pain ratings by expectation and uncertainty: behavioral characteristics and anticipatory neural correlates. *Pain* 135 240–250 10.1016/j.pain.2007.05.022 17614199

[B9] Burgess G. Grogan S. Burwitz L. (2006). Effects of a 6-week aerobic dance intervention on body image and physical self-perceptions in adolescent girls. *Body Image* 3 57–66 10.1016/j.bodyim.2005.10.005 18089209

[B10] Cooper P. J. Taylor M. J. Cooper Z. Fairburn C. G. (1987). The development and validation of the body shape questionnaire. *Int. J. Eat Disord.* 6 485–494 10.1002/1098-108X(198707)6:4<485::AID-EAT2260060405>3.0.CO;2-O

[B11] Diers M. Zieglgänsberger W. Trojan J. Drevensek A. M. Erhardt-Raum G. Flor H. (2013). Site-specific visual feedback reduces pain perception. *Pain* 154 890–896 10.1016/j.pain.2013.02.022 23582151

[B12] Drummond P. D. Willox M. (2013). Painful effects of auditory startle, forehead cooling and psychological stress in patients with fibromyalgia or rheumatoid arthritis. *J. Psychosom. Res.* 74 378–383 10.1016/j.jpsychores.2013.01.011 23597324

[B13] Eisenberger N. I. Master S. L. Inagaki T. K. Taylor S. E. Shirinyan D. Lieberman M. D. (2011). Attachment figures activate a safety signal-related neural region and reduce pain experience. *Proc. Natl. Acad. Sci. U.S.A.* 108 11721–11726 10.1073/pnas.1108239108 21709271 PMC3136329

[B14] Eshkevari E. Rieger E. Longo M. R. Haggard P. Treasure J. (2012). Increased plasticity of the bodily self in eating disorders. *Psychol. Med.* 42 819–828 10.1017/S0033291711002091 22017964

[B15] Friederich H. C. Brooks S. Uher R. Campbell I. C. Giampietro V. Brammer M. (2010). Neural correlates of body dissatisfaction in anorexia nervosa. *Neuropsychologia* 48 2878–2885 10.1016/j.neuropsychologia.2010.04.036 20553738

[B16] Ghaderi A. Scott B. (2004). The reliability and validity of the Swedish version of the body shape questionnaire. *Scand. J. Psychol.* 45 319–324 10.1111/j.1467-9450.2004.00411.x 15281921

[B17] Höfle M. Hauck M. Engel A. K. Senkowski D. (2010). Pain processing in multisensory environments. *e-Neuroforum* 1 23–28 10.1007/s13295-010-0004-z

[B18] Höfle M. Hauck M. Engel A. K. Senkowski D. (2012). Viewing a needle pricking a hand that you perceive as yours enhances unpleasantness of pain. *Pain* 153 1074–1081 10.1016/j.pain.2012.02.010 22520059

[B19] Kennett S. Taylor-Clarke M. Haggard P. (2001). Non informative vision improves the spatial resolution of touch in humans. *Curr. Biol.* 11 1188–1191 10.1016/S0960-9822(01)00327-X 11516950

[B20] Koyama T. McHaffie J. G. Laurienti P. J. Coghill R. C. (2005). The subjective experience of pain: where expectations become reality. *Proc. Natl. Acad. Sci. U.S.A.* 102 12950–12955 10.1073/pnas.0408576102 16150703 PMC1200254

[B21] Lewis J. S. Kersten P. McCabe C. S. McPherson K. M. Blake D. R. (2007). Body perception disturbance: a contribution to pain in complex regional pain syndrome (CRPS). *Pain* 133 111–119 10.1016/j.pain.2007.03.013 17509761

[B22] Longo M. R. Betti V. Aglioti S. M. Haggard P. (2009). Visually induced analgesia: seeing the body reduces pain. *J. Neurosci.* 29 12125–12130 10.1523/JNEUROSCI.3072-09.2009 19793970 PMC6666129

[B23] Longo M. R. Haggard P. (2011). Weber's illusion and body shape: anisotropy of tactile size perception on the hand. *J. Exp. Psychol. Hum. Percept. Perform.* 37 720–726 10.1037/a0021921 21480744

[B24] Longo M. R. Iannetti G. D. Mancini F. Driver J. Haggard P. (2012). Linking pain and the body: neural correlates of visually induced analgesia. *J. Neurosci.* 32 2601–2607 10.1523/JNEUROSCI.4031-11.2012 22357844 PMC6621879

[B25] Luomajoki H. Moseley G. L. (2011). Tactile acuity and lumbopelvic motor control in patients with back pain and healthy controls. *Br. J. Sports Med.* 45 437–440 10.1136/bjsm.2009.060731 19553222

[B26] Mancini F. Longo M. R. Kammers M. P. Haggard P. (2011). Visual distortion of body size modulates pain perception. *Psychol. Sci.* 22 325–330 10.1177/0956797611398496 21303990

[B27] Martini M. Perez-Marcos D. Sanchez-Vives M. V. (2013). What color is my arm? Changes in skin color of an embodied virtual arm modulates pain threshold. *Front. Hum. Neurosci.* 7:438 10.3389/fnhum.2013.00438 PMC3728482 23914172

[B28] McCabe C. S. Cohen H. Blake D. R. (2007). Somaesthetic disturbances in fibromyalgia are exaggerated by sensory motor conflict: implications for chronicity of the disease? *Rheumatology* (Oxford) 46 1587–1592 10.1093/rheumatology/kem204 17767000

[B29] McCabe C. S. Haigh R. C. Halligan P. W. Blake D. R. (2005). Simulating sensory-motor incongruence in healthy volunteers: implications for a cortical model of pain. *Rheumatology* (Oxford) 44 509–516 10.1093/rheumatology/keh529 15644392

[B30] Meagher M. W. Arnau R. C. Rhudy J. L. (2001). Pain and emotion: effects of affective picture modulation. *Psychosom. Med.* 63 79–90 11211069 10.1097/00006842-200101000-00010

[B31] Miyake Y. Okamoto Y. Onoda K. Kurosaki M. Shirao N. Okamoto Y. (2010). Brain activation during the perception of distorted body images in eating disorders. *Psychiatry Res.* 181 183–192 10.1016/j.pscychresns.2009.09.001 20153150

[B32] Moberg E. (1990). Two-point discrimination test. A valuable part of hand surgical rehabilitation, e.g. in tetraplegia. *Scand. J. Rehabil. Med.* 22 127–134 2244189

[B33] Mohr H. M. Röder C. Zimmermann J. Hummel D. Negele A. Grabhorn R. (2011). Body image distortions in bulimia nervosa: investigating body size overestimation and body size satisfaction by fMRI. *Neuroimage* 56 1822–1831 10.1016/j.neuroimage.2011.02.069 21362488

[B34] Moseley G. L. (2005). Distorted body image in complex regional pain syndrome. *Neurology* 65 773 10.1212/01.wnl.0000174515.07205.11 16157921

[B35] Moseley G. L. Flor H. (2012). Targeting cortical representations in the treatment of chronic pain: a review. *Neurorehabil. Neural Repair* 26 646–652 10.1177/1545968311433209 22331213

[B36] Moseley G. L. Gallace A. Spence C. (2012). Bodily illusions in health and disease: physiological and clinical perspectives and the concept of a cortical `body matrix'. *Neurosci. Biobehav. Rev.* 36 34–46 10.1016/j.neubiorev.2011.03.013 21477616

[B37] Moseley G. L. Parsons T. J. Spence C. (2008a). Visual distortion of a limb modulates the pain and swelling evoked by movement. *Curr. Biol.* 18 1047–1048 10.1016/j.cub.2008.09.031 19036329

[B38] Moseley G. L. Zalucki N. M. Wiech K. (2008b). Tactile discrimination, but not tactile stimulation alone, reduces chronic limb pain. *Pain* 137 600–608 10.1016/j.pain.2007.10.021 18054437

[B39] Moseley G. L. Wiech K. (2009). The effect of tactile discrimination training is enhanced when patients watch the reflected image of their unaffected limb during training. *Pain* 144 314–319 10.1016/j.pain.2009.04.030 19501965

[B40] Peltz E. Seifert F. Lanz S. Müller R. Maihöfner C. (2011). Impaired hand size estimation in CRPS. *J. Pain* 12 1095–1091 10.1016/j.jpain.2011.05.001 21741321

[B41] Ramachandran V. S. Brang D. McGeoch P. D. (2009). Size reduction using mirror visual feedback (MVF) reduces phantom pain. *Neurocase* 15 357–360 10.1080/13554790903081767 19657972

[B42] Serino A. Farne A. Rinaldesi M. L. Haggard P. Ladavas E. (2007). Can vision of the body ameliorate impaired somatosensory function? *Neuropsychologia* 45 1101–1107 10.1016/j.neuropsychologia.2006.09.013 17101158

[B43] Smeets M. A. Klugkist I. G. Rooden S. V. Anema H. A. Postma A. (2009). Mental body distance comparison: a tool for assessing clinical disturbances in visual body image. *Acta Psychol. (Amst.)* 132 157–165 10.1016/j.actpsy.2009.03.011 19406374

[B44] Spangler D. L. Allen M. D. (2012). An fMRI investigation of emotional processing of body shape in bulimia nervosa. *Int. J. Eat Disord.* 45 17–25 10.1002/eat.20899 21312206

[B45] Stanton T. R. Lin C. W. Bray H. Smeets R. J. Taylor D. Law R. Y. (2013). Tactile acuity is disrupted in osteoarthritis but is unrelated to disruptions in motor imagery performance. *Rheumatology* 52 1509–1519 10.1093/rheumatology/ket139 23661429

[B46] Taylor-Clarke M. Jacobsen P. Haggard P. (2004). Keeping the world a constant size: object constancy in human touch. *Nat. Neurosci.* 7 219–220 10.1038/nn1199 14966526

[B47] Tracey I. Mantyh P. W. (2007). The cerebral signature for pain perception and its modulation. *Neuron* 55 377–391 10.1016/j.neuron.2007.07.012 17678852

[B48] Traverso A. Ravera G. Lagattolla V. Testa S. Adami G. F. (2000). Weight loss after dieting with behavioral modification for obesity: the predicting efficiency of some psychometric data. *Eat Weight Disord.* 5 102–107 10.1007/BF03327485 10941608

[B49] Vartiainen N. Kirveskari E. Kallio-Laine K. Kalso E. Forss N. (2009). Cortical reorganization in primary somatosensory cortex in patients with unilateral chronic pain. *J. Pain* 10 854–859 10.1016/j.jpain.2009.02.006 19638329

[B50] Villemure C. Slotnick B. M. Bushnell M. C. (2003). Effects of odors on pain perception: deciphering the roles of emotion and attention. *Pain* 106 101–108 10.1016/S0304-3959(03)00297-5 14581116

[B51] Wade T. D. Wilkinson J. Ben-Tovim D. (2003). The genetic epidemiology of body attitudes, the attitudinal component of body image in women. *Psychol. Med.* 33 1395–1405 10.1017/S0033291703008572 14672248

[B52] Wand B. M. O’Connell N. E. Di Pietro F. Bulsara M. (2011). Managing chronic nonspecific low back pain with a sensorimotor retraining approach: exploratory multiple-baseline study of 3 participants. *Phys. Ther.* 91 535–546 10.2522/ptj.20100150 21350034

[B53] Wand B. M. Tulloch V. M. George P. J. Smith A. J. Goucke R. O’Connell N. E. (2012). Seeing it helps: movement-related back pain is reduced by visualization of the back during movement. *Clin. J. Pain* 28 602–608 10.1097/AJP.0b013e31823d480c 22699134

[B54] Welch E. Lagerström M. Ghaderi A. (2012). Body shape questionnaire: psychometric properties of the short version (BSQ-8C) and norms from the general Swedish population. *Body Image* 9 547–550 10.1016/j.bodyim.2012.04.009 22721875

[B55] Yarnitsky D. Sprecher E. Zaslansky R. Hemli J. A. (1995). Heat pain thresholds: normative data and repeatability. *Pain* 60 329–332 10.1016/0304-3959(94)00132-X 7596629

[B56] Younger J. Aron A. Parke S. Chatterjee N. Mackey S. (2010). Viewing pictures of a romantic partner reduces experimental pain: involvement of neural reward systems. *PLoS ONE* 5:e13309 10.1371/journal.pone.0013309 PMC2954158 20967200

